# Filamentous Giant Beggiatoaceae from the Guaymas Basin Are Capable of both Denitrification and Dissimilatory Nitrate Reduction to Ammonium

**DOI:** 10.1128/AEM.02860-17

**Published:** 2018-07-17

**Authors:** Charles A. Schutte, Andreas Teske, Barbara J. MacGregor, Verena Salman-Carvalho, Gaute Lavik, Philipp Hach, Dirk de Beer

**Affiliations:** a Max Planck Institute for Marine Microbiology, Bremen, Germany; b Department of Marine Sciences, University of North Carolina at Chapel Hill, Chapel Hill, North Carolina, USA; University of Illinois at Urbana-Champaign

**Keywords:** DNRA, denitrification, nitrogen cycle, biogeochemistry, marine microbiology

## Abstract

Filamentous large sulfur-oxidizing bacteria (FLSB) of the family Beggiatoaceae are globally distributed aquatic bacteria that can control geochemical fluxes from the sediment to the water column through their metabolic activity. FLSB mats from hydrothermal sediments of Guaymas Basin, Mexico, typically have a “fried-egg” appearance, with orange filaments dominating near the center and wider white filaments at the periphery, likely reflecting areas of higher and lower sulfide fluxes, respectively. These FLSB store large quantities of intracellular nitrate that they use to oxidize sulfide. By applying a combination of ^15^N-labeling techniques and genome sequence analysis, we demonstrate that the white FLSB filaments were capable of reducing their intracellular nitrate stores to both nitrogen gas and ammonium by denitrification and dissimilatory nitrate reduction to ammonium (DNRA), respectively. On the other hand, our combined results show that the orange filaments were primarily capable of DNRA. Microsensor profiles through a laboratory-incubated white FLSB mat revealed a 2- to 3-mm vertical separation between the oxic and sulfidic zones. Denitrification was most intense just below the oxic zone, as shown by the production of nitrous oxide following exposure to acetylene, which blocks nitrous oxide reduction to nitrogen gas. Below this zone, a local pH maximum coincided with sulfide oxidation, consistent with nitrate reduction by DNRA. The balance between internally and externally available electron acceptors (nitrate) and electron donors (reduced sulfur) likely controlled the end product of nitrate reduction both between orange and white FLSB mats and between different spatial and geochemical niches within the white FLSB mat.

**IMPORTANCE** Whether large sulfur bacteria of the family Beggiatoaceae reduce NO_3_^−^ to N_2_ via denitrification or to NH_4_^+^ via DNRA has been debated in the literature for more than 25 years. We resolve this debate by showing that certain members of the Beggiatoaceae use both metabolic pathways. This is important for the ecological role of these bacteria, as N_2_ production removes bioavailable nitrogen from the ecosystem, whereas NH_4_^+^ production retains it. For this reason, the topic of environmental controls on the competition for NO_3_^−^ between N_2_-producing and NH_4_^+^-producing bacteria is of great scientific interest. Recent experiments on the competition between these two types of microorganisms have demonstrated that the balance between electron donor and electron acceptor availability strongly influences the end product of NO_3_^−^ reduction. Our results suggest that this is also the case at the even more fundamental level of enzyme system regulation within a single organism.

## Abstract (precis)

Whether large sulfur bacteria of the family Beggiatoaceae reduce NO_3_^−^ to N_2_ via denitrification or to NH_4_^+^ via DNRA has been debated in the literature for more than 25 years. We resolve this debate by showing that certain members of the Beggiatoaceae use both metabolic pathways. This is important for the ecological role of these bacteria, as N_2_ production removes bioavailable nitrogen from the ecosystem, whereas NH_4_^+^ production retains it. For this reason, the topic of environmental controls on the competition for NO_3_^−^ between N_2_-producing and NH_4_^+^-producing bacteria is of great scientific interest. Recent experiments on the competition between these two types of microorganisms have demonstrated that the balance between electron donor and electron acceptor availability strongly influences the end product of NO_3_^−^ reduction. Our results suggest that this is also the case at the even more fundamental level of enzyme system regulation within a single organism.

## INTRODUCTION

The family Beggiatoaceae includes a diverse group of large sulfur-oxidizing bacteria, like Thioploca, Beggiatoa, and Thiomargarita spp., that employ an equally diverse set of physiological and metabolic adaptations to inhabit a wide range of niches in benthic aquatic environments (1, 2). A subset of this family, the filamentous large sulfur-oxidizing bacteria (FLSB), are generally described as motile vacuolated filament-forming chemolithoautotrophs and mixotrophs that form characteristic mats on the seafloor (1–3). Filaments can reach over 100 μm in diameter and accumulate and store up to several hundred millimolar NO_3_^−^ in internal vacuoles (4). FLSB mats are typically found on sediments with low O_2_ content in the overlying water and a substantial HS^−^ flux from deeper sediment layers (5). Filaments use their gliding motility to move between oxic and sulfidic zones in the sediment, mostly residing where both O_2_ and HS^−^ concentrations are very low (6–8). Under anoxic conditions, FLSB use NO_3_^−^ stored in their vacuoles to oxidize HS^−^ to elemental sulfur (S^0^), which they also store internally. Near the sediment surface, they recharge their NO_3_^−^ stores and use O_2_ or NO_3_^−^ to oxidize their stored S^0^ to SO_4_^2−^ that is excreted (6).

There is an ongoing debate as to whether FLSB reduce their internally stored NO_3_^−^ to N_2_ by denitrification or to NH_4_^+^ by DNRA. Freshwater FLSB mats dominated by Beggiatoa spp. were shown to reduce NO_3_^−^ to N_2_ and NH_4_^+^, while the isolated freshwater type strain of Beggiatoa alba reduces NO_3_^−^ to NH_4_^+^ only (9, 10). Whole-sediment studies of coastal FLSB mats suggested that DNRA is the dominant pathway of NO_3_^−^ reduction (6, 11), while Arctic marine FLSB showed N_2_O accumulation after the addition of acetylene, indicating denitrification (12). Targeted studies trying to solve this dispute are complicated by the fact that no cultures of NO_3_^−^-storing FLSB are available.

Genomic information from FLSB that can be used to infer their metabolic pathways is currently limited to several partial genomes of marine filaments (12–15), reconstructed metagenomes (16), and cultured strains (17–19). Complete pathways have not been identified for either denitrification or DNRA. Putative genes for the dissimilatory nitrate reductase (NxrA/NarG) necessary for both pathways have been identified in all marine FLSB, along with the nitrite (NO_2_^−^) and nitric oxide (NO) reductases (NirS and NorB, respectively) required for denitrification. However, genes encoding the enzymes for the terminal steps in both pathways, NO_2_^−^ reduction to NH_4_^+^ in DNRA (catalyzed by NirB) and nitrous oxide reduction to N_2_ in denitrification (catalyzed by NosZ), could often not be identified. Therefore, genomic information has been insufficient to resolve the debate about the usage of NO_3_^−^ reduction pathways in large vacuolated FLSB.

Motile FLSB traveling between oxic and sulfidic sediment zones are exposed to extreme redox shifts. We hypothesized that these motile FLSB might be capable of both denitrification and DNRA, with the activity of each process shifting in response to the filament's position relative to O_2_ and HS^−^ gradients. To test this hypothesis, we experimented with white and orange FLSB mats that are morphologically and phylogenetically distinct (20) and occupy different zones within the highly dynamic hydrothermal sediments of the Guaymas Basin (21). Orange FLSB mats dominate in sediments with strong hydrothermal flow where HS^−^ supply is maximal and often reaches close to the surface (20). White FLSB mats, on the other hand, typically dominate at the periphery of these regions, where hydrothermal activity and HS^−^ fluxes are attenuated. The resulting “fried-egg” pattern, with an orange FLSB mat surrounded by a white FLSB mat, is consistently observed in organisms from the Guaymas Basin (21). We incubated FLSB filaments with [^15^N]NO_3_^−^ and tracked its transformation into both [^15^N]NH_4_^+^ and [^15^N]N_2_. Microsensor measurements revealed the vertical distribution of denitrification through the FLSB mat relative to O_2_, HS^−^, and pH profiles, and the depth of maximum DNRA activity could also be inferred. This was complemented with an assessment of the genes potentially encoding the denitrification and DNRA pathways in orange and white FLSB filaments.

## RESULTS

### [^15^N]NO_3_^−^ incubations.

**FIG 1:**
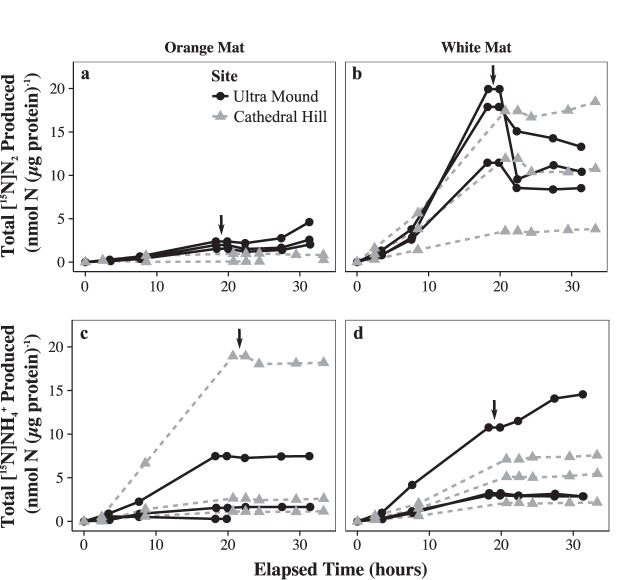
Time series of cumulative [^15^N]NO_3_^−^ reduction to [^15^N]N_2_ (a and b) and to [^15^N]NH_4_^+^ (c and d), normalized to protein content for each of the six orange (a and c) and six white (b and d) laboratory-incubated FLSB mat pieces. Each line represents an individual mat piece. The vertical black arrows indicate when the FLSB mats were destroyed at around 20 h of incubation.

Laboratory-incubated white Guaymas Basin FLSB mats performed denitrification and DNRA simultaneously. ^15^N accumulated in both the N_2_ and NH_4_^+^ pools when cleaned filaments were incubated with [^15^N]NO_3_^−^ as the substrate, though [^15^N]N_2_ was consistently produced at a higher rate than [^15^N]NH_4_^+^ (Fig. 1 and Table 1). After the FLSB filaments were destroyed, [^15^N]N_2_ and [^15^N]NH_4_^+^ production ceased or decreased by at least a factor of ∼2 (Fig. 1 and Table 1). Thus, most of the observed NO_3_^−^ reduction was driven by the activity of the FLSB filaments and not by their smaller microbial epibionts. Orange mats produced less [^15^N]N_2_ than white mats, and this production was driven entirely by epibionts in five out of six replicates. Notably, the [^15^N]N_2_ accumulation rate remained effectively unchanged before and after the filament destruction treatment in these orange mats (Table 1), indicating that epibiont denitrification activity was unaffected by this treatment. On the other hand, orange mats produced [^15^N]NH_4_^+^ at rates similar to or higher than the white mats (Fig. 1 and Table 1), and [^15^N]NH_4_^+^ production decreased by at least a factor of 7 following filament destruction, indicating that this NO_3_^−^ reduction pathway was driven by the orange FLSB. Taken together, these data demonstrate that DNRA was the only significantly active NO_3_^−^ reduction pathway in FLSB from orange mats, and both pathways were active in FLSB from white mats, with denitrification occurring at a higher rate than DNRA.

**TABLE 1:** Rates of [^15^N]NO_3_^−^ reduction to [^15^N]N_2_ and [^15^N]NH_4_^+^ from each of the six orange and six white laboratory-incubated FLSB mat pieces

Mat	Site^*a*^	Treatment^*b*^	Protein content (μg · liter^−1^)	Production rate (pmol N · [μg protein]^−1^ · h^−1^)^*c*^					
				[^15^N]N_2_			[^15^N]NH_4_^+^		
				Whole mat	Epibionts	FLSB	Whole mat	Epibionts	FLSB
White	UM	SW	4.4	652 ± 94	0 ± 0	652 ± 94	168 ± 9	0 ± 0	168 ± 9
		SW	2	1,140 ± 234	106 ± 145	1,034 ± 275	183 ± 19	0 ± 0	183 ± 19
		+HS^−^	1.2	1,016 ± 157	0 ± 0	1,016 ± 157	611 ± 40	347 ± 109	264 ± 116
	CH	SW	8.2	583 ± 39	40 ± 29	543 ± 49	251 ± 22	47 ± 10	204 ± 24
		+HS^−^	23	174 ± 4	45 ± 8	129 ± 9	103 ± 9	14 ± 1	90 ± 9
		+DOC	13.9	847 ± 55	195 ± 31	651 ± 63	346 ± 32	25 ± 10	321 ± 33
Orange	UM	SW	19	110 ± 15	115 ± 59	0 ± 61	6 ± 23	21 ± 10	0 ± 25
		SW	15	86 ± 12	91 ± 35	0 ± 37	87 ± 12	1 ± 0	86 ± 12
		+HS^−^	9.7	133 ± 15	263 ± 101	0 ± 102	419 ± 37	24 ± 8	395 ± 38
	CH	SW	2.5	0 ± 0	0 ± 0	0 ± 0	949 ± 61	17 ± 0	932 ± 61
		+HS^−^	6.9	0 ± 0	0 ± 0	0 ± 0	134 ± 18	18 ± 3	116 ± 18
		+DOC	4.4	45 ± 12	0 ± 0	45 ± 12	54 ± 4	7 ± 4	47 ± 6

a Sampling locations were Ultra Mound (UM) and Cathedral Hill (CH); more details can be found in Table S1 and Fig. S1.

b Treatments were seawater only (SW), seawater + HS^−^ (+HS^−^), and seawater + dissolved organic carbon (+DOC) in the form of acetate.

c Rates were calculated as the slope of the linear regression of the time points displayed in Fig. 1. Uncertainty in these rates was calculated as the standard error of the slope. The “whole mat” rate is the rate calculated from the first phase of each incubation, when FLSB filaments were intact. The “epibionts” rate is the rate calculated from the second phase of each incubation, after the FLSB filaments were destroyed. The “FLSB” rate is the whole mat rate minus the epibionts rate. This rate represents the activity of only the FLSB filaments. In many cases, the whole mat and FLSB rates are similar, because there was very little activity following filament destruction, demonstrating that the filaments were responsible for most of the total mat activity.

Acetate and HS^−^ additions did not influence nitrogen conversion rates consistently between different sampling sites. At Ultra Mound, both white and orange FLSB [^15^N]NH_4_^+^ production rates were highest in sulfide-added treatments. At Cathedral Hill, both white and orange FLSB [^15^N]N_2_ production rates were highest in acetate-added treatments (Table 1). Filaments from both white and orange mats were also incubated with [^15^N]NH_4_^+^, and no ^29^N_2_ production was observed (data not shown). Therefore, the N_2_ production observed in other treatments was not driven by organisms that perform anammox, a process associated with other FLSB of the Beggiatoaceae, i.e., bundle-forming Thioploca-like filaments and their sheath epibionts (22).

### Microsensor measurements.

**FIG 2:**
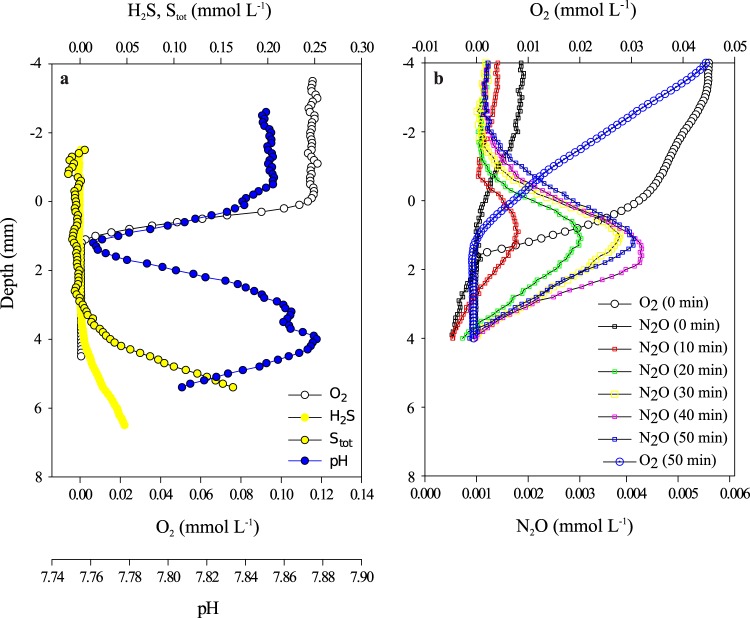
Microsensor profiles in a white FLSB mat. The top of the mat was carefully observed and is located at depth 0.0 cm in the profiles. (a and b) Oxygen, H_2_S, total sulfides (S_tot_), and pH microsensor profiles (a), and oxygen and N_2_O microsensor profiles (b) measured at 10-min intervals from 0 to 50 min following the addition of acetylene.

In the white FLSB mat that survived transport back to Germany, O_2_ diffusing from seawater into the mat was consumed in the top 1 mm (Fig. 2a). The depth where O_2_ disappeared coincided with a pH minimum. HS^−^ diffused upwards from a thin layer of sediment at the bottom of the container and was consumed at around 3 mm depth. There was a 2-mm-wide gap between the first and the third millimeter, in which neither O_2_ nor HS^−^ was detectable. A pH maximum was observed at around 4 mm. Following the addition of 20% acetylene to the seawater medium of the same white FLSB mat, N_2_O was produced throughout the mat within 10 min, and production persisted for at least 30 min. Maximum N_2_O production occurred at a depth of around 1 mm inside the mat, just below the zone where O_2_ disappeared (Fig. 2b). In a second approach, N_2_O production in white FLSB mats was investigated in batch incubations. These mats also produced N_2_O after acetylene was added, and N_2_O production ceased after the FLSB filaments were destroyed (see Fig. S4 in the supplemental material). We conclude that the FLSB filaments, and not the associated smaller microorganisms, produced the N_2_O.

### Nitrate reduction genes in FLSB genomes.

**FIG 3:**
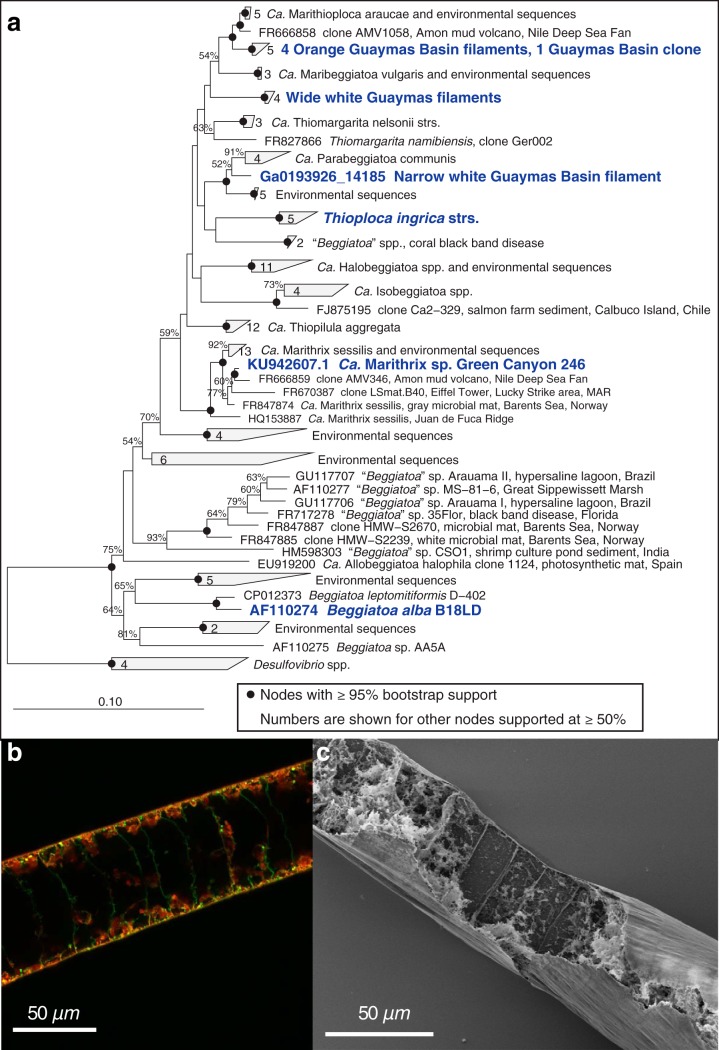
16S rRNA gene distance phylogeny of Guaymas Basin Beggiatoaceae (a). Species analyzed in Table 1 and others relevant to this study are shown in boldface. The two morphologically distinct Guaymas filaments (orange-pigmented and wide white) form two separate clusters. Each cluster contains sequences obtained from different mats during different sampling campaigns. (b) Combined fluorescein isothiocyanate (green) and Nile Red (red) staining of the white filaments (see Supplemental Methods for details) indicates the presence of a large internal vacuole in each cell. Green stain shows the location of the cytoplasm, red stain shows lipid layers and droplets, and yellow shows areas of overlap between green and red stains. The large unstained area in the center of each cell is a negative image of the central aqueous vacuole. (c) Scanning electron microscopy of a manually opened white filament (see Supplemental Methods for details) likewise showed that major parts of the biovolume of the filaments are empty, representing the internal vacuole.

The orange filament genome (13) includes putative genes for dissimilatory nitrate reduction to nitrite via either NxrA/NarG or periplasmic NapF (filled squares in Table 2). However, no gene for NO_2_^−^ reductase (NirB) in the DNRA pathway was found (open squares in Table 2). Within the denitrification pathway, we could not identify genes for all subunits of the nitrous oxide reductase (Nos), for most subunits of the NO-forming nitrite reductase (NirS), or for the nitric oxide reductase (Nor) (Table 2). In contrast, the genome from unpigmented white Guaymas Basin filaments (16) (Fig. 3) has almost-complete sets of genes for both DNRA and denitrification (Table 2).

**TABLE 2:**
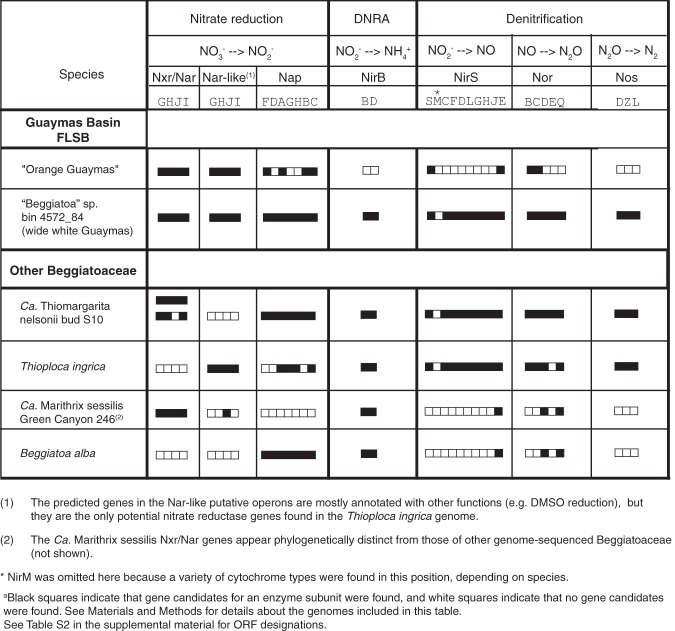
Overview of nitrate reduction pathway genes predicted for representative large sulfur bacteria^*a*^

### Phylogeny and morphology of FLSB filaments.

Narrow orange and wide white Guaymas Basin filaments are phylogenetically distinct, as shown by analyzing the 16S rRNA gene sequences obtained from individual filaments collected over several sampling campaigns (Fig. 3a). Consistent with morphological descriptions of other FLSB, we could detect a thin layer of membrane and cytoplasm surrounding a large vacuole that took up most of the cell volume in the multicellular wide white filaments (Fig. 3b and c).

## DISCUSSION

### Simultaneous denitrification and DNRA activity in FLSB mats.

We demonstrated that lab-incubated white FLSB mats from Guaymas Basin can reduce NO_3_^−^ to both N_2_ via denitrification and to NH_4_^+^ via DNRA (Fig. 1 and Table 1). We further showed that the bulk turnover in both denitrification and DNRA could be attributed to the FLSB filaments and not the smaller mat-associated microorganisms (epibionts). Although genome analyses of different types of FLSB indicate that genes for denitrification and DNRA can in some cases occur jointly in the same organism (Table 2), previous studies using whole mats only showed that the presence of the FLSB promoted either denitrification (9, 12) or DNRA (6, 11).

The filaments that form the differently colored FLSB mats from Guaymas Basin can be sorted into several size classes (5, 20) and potentially harbor several phylotypes based on 16S rRNA gene sequencing (20). Filaments of various diameters were also observed in the mats used in this study. Combined with earlier samples, each morphogroup (medium-sized orange, wide white, and narrow white filaments) forms a distinct monophyletic cluster among other vacuolated, marine filamentous, and nonfilamentous LSB in the family Beggiatoaceae (Fig. 3). Here, we reanalyzed a genome that was originally reconstructed from a sediment-derived metagenome (16). Based on 16S rRNA gene sequence analysis, we confidently assign it to represent the genetic potential of the wide white filaments that also make up the bulk of white FLSB mats. We identified the complete pathways for both denitrification and DNRA within this genome (Table 2) and conclude that these filaments had the genetic potential to carry out both processes as observed in the stable isotope incubations (Fig. 1).

In lab-incubated FLSB mats dominated by orange filaments, DNRA was the only NO_3_^−^ reduction activity that could be associated consistently with the FLSB filaments (Fig. 1). However, we could not find complete DNRA or denitrification pathways in the genome of a single orange filament (Table 2), reemphasizing that negative genomic results carry no proof. Given the estimated 98% completeness of this genome (18), it is possible that these genes reside in the as-yet-unsequenced portion of the genome. It is also possible that these filaments carry out DNRA by a novel mechanism. For example, the octaheme cytochrome that gives the orange filaments their color was demonstrated to reduce nitrite to ammonia *in vitro* (23), giving it the potential to replace the traditional NirB nitrite reductase enzyme in the DNRA pathway. Sequence database comparisons show that this protein falls within a large group of octaheme cytochromes annotated as hydrazine or hydroxylamine oxidases. These functions are part of the nitrification (24) and anammox (25) pathways that are not present in Beggiatoaceae (13). Thus, the cytochrome in the orange FLSB likely does not oxidize hydrazine or hydroxylamine but may instead act as a nitrite reductase in the DNRA pathway.

### Vertical stratification of NO_3_^−^ reduction pathways in FLSB mats.

The sigmoidal pH profile measured in a lab-incubated white FLB mat (Fig. 2a) shows that different biogeochemical processes drove a local pH minimum at around 1 mm depth (coincident with complete O_2_ reduction) and a local pH maximum at around 3 mm depth (coincident with complete HS^−^ oxidation). The shallow pH minimum is consistent with the oxidation of intracellularly stored S^0^ using either O_2_ or NO_3_^−^ as the electron acceptor, regardless of the NO_3_^−^ reduction pathway.

$${\text{S}}^{0}+1.5\;{\text{O}}_{2}+{\text{H}}_{2}\text{O}\to {{\text{SO}}_{4}}^{2-}+4\;{\text{H}}^{+}$$

$${\text{S}}^{0}+0.75\;{{\text{NO}}_{3}}^{-}+1.75\;{\text{H}}_{2}\text{O}\to {{\text{SO}}_{4}}^{2-}+0.75\;{{\text{NH}}_{4}}^{+}+0.5\;{\text{H}}^{+}$$

$${\text{S}}^{0}+1.2\;{{\text{NO}}_{3}}^{-}+0.4\;{\text{H}}_{2}\text{O}\to {{\text{SO}}_{4}}^{2-}+0.6\;{\text{N}}_{2}+0.8\;{\text{H}}^{+}$$

N_2_O accumulated most rapidly at this depth following the addition of acetylene (Fig. 2b), indicating that denitrification was most active there.

The deep pH maximum is consistent with HS^−^ oxidation to either SO_4_^2−^ or S^0^ using NO_3_^−^ as an electron acceptor, regardless of whether NO_3_^−^ was reduced to N_2_ or NH_4_^+^:

$${\text{HS}}^{-}+0.25\;{{\text{NO}}_{3}}^{-}+1.5\;{\text{H}}^{+}\to {\text{S}}^{0}+0.25\;{{\text{NH}}_{4}}^{+}+0.75\;{\text{H}}_{2}\text{O}$$

$${\text{HS}}^{-}+{{\text{NO}}_{3}}^{-}+{\text{H}}^{+}+{\text{H}}_{2}\text{O}\to {{\text{SO}}_{4}}^{2-}+{{\text{NH}}_{4}}^{+}$$

$${\text{HS}}^{-}+0.4\;{{\text{NO}}_{3}}^{-}+1.4\;{\text{H}}^{+}\to {\text{S}}^{0}+0.2\;{\text{N}}_{2}+1.2\;{\text{H}}_{2}\text{O}$$

$${\text{HS}}^{-}+1.6\;{{\text{NO}}_{3}}^{-}+0.6\;{\text{H}}^{+}\to {{\text{SO}}_{4}}^{2-}+0.8\;{\text{N}}_{2}+0.8\;{\text{H}}_{2}\text{O}$$

Much less N_2_O was produced at this depth, indicating a limited role for denitrification. Since the pH minimum and maximum were of similar magnitude, and DNRA and denitrification produce a similar number of protons per NO_3_^−^ reduced (6 and 3.5, respectively), it is probable that DNRA was the more important NO_3_^−^ reduction pathway deep in the mat. Furthermore, given that white mats reduced more NO_3_^−^ to N_2_ than to NH_4_^+^ overall (Fig. 1 and Table 1), it is likely that denitrification was the more important pathway near the mat surface where maximum N_2_O production was observed.

Using microsensors, we found that denitrification and DNRA were most active in different geochemical and spatial niches within a white FLSB mat. We conclude that in FLSB mats with vertical separation between the O_2_ and HS^−^ depletion zones, more FLSB filaments couple HS^−^ oxidation to S^0^ with DNRA than denitrification in deeper layers of the mat where HS^−^ is present, and more filaments couple the oxidation of internally stored S^0^ to SO_4_^2−^ with denitrification than DNRA near the surface of the mat where HS^−^ is absent. According to this model, the two NO_3_^−^ reduction pathways are active simultaneously within a single mat but are most active at different depths. Genetic evidence suggests that individual organisms of the Beggiatoaceae have the potential to perform denitrification and DNRA (Table 2), but this remains to be experimentally demonstrated for large vacuolated mat-forming FLSB, such as those used in this study. Our data establish the physicochemical characteristics of a mat environment and provide the physiological setting for differential gene expression. Motile FLSB filaments travel between different redox zones (8) and may activate their NO_3_^−^ reduction pathways at different times depending on their position within the mat relative to O_2_, HS^−^, and pH gradients.

### Controls of N_2_ versus NH_4_^+^ production in FLSB mats.

**FIG 4:**
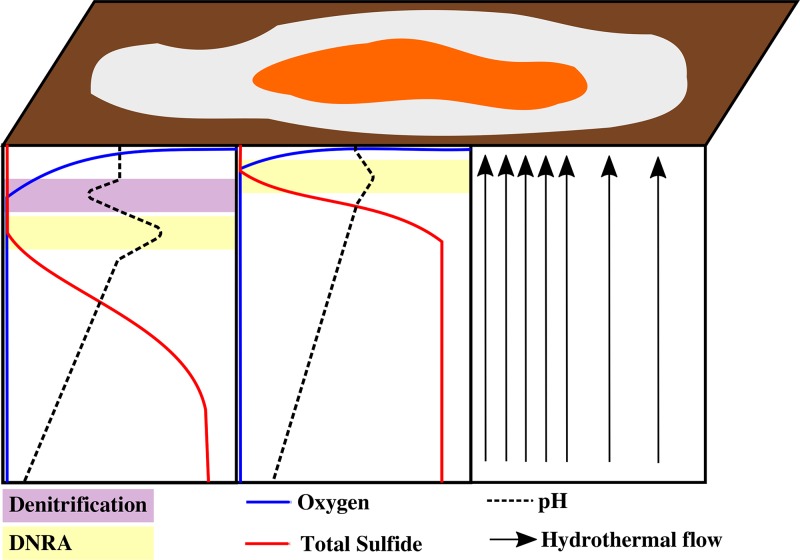
Conceptual diagram of a Guaymas Basin FLSB mat with an orange center and a white periphery, surrounded by bare sediment (brown). The intensity of hydrothermal flow is represented by the density of black arrows. They show more intense hydrothermal flow under the center of the mat where the orange FLSB dominate and reduced hydrothermal flow under the mat periphery where white FLSB dominate. Idealized oxygen, total sulfide, and pH gradients in sediment beneath the mat center and periphery are displayed with arbitrary depth and concentration units. The purple and yellow zones show the relative positions of the dominant NO_3_^−^ reduction pathways, denitrification and DNRA, respectively.

The white mats performed both DNRA and denitrification, but denitrification generally occurred at a higher rate. In contrast, DNRA rates were much higher than denitrification rates in orange mats, and evidence indicates that the orange FLSB filaments performed DNRA only. These trends were reproducible across mats from different sampling locations in spite of between-mat variability in absolute rates (Table 1). This physiological difference appears to correlate with different habitat preferences and electron donor availability (Fig. 4). The orange FLSB dominate at the centers of hydrothermal sediments, where HS^−^ supply is maximal and often reaches close to the surface (20). White filaments typically dominate at the periphery of these regions, where hydrothermal activity and HS^−^ fluxes are attenuated. The resulting “fried-egg” pattern is consistently observed in Guaymas Basin FLSB mats (21), indicating that these ecophysiological preferences are linked to these genetically and morphologically distinct FLSB types in a consistent manner and do not merely represent different gene expression patterns or behavioral adaptations in a highly flexible population (20).

In hydrothermal environments, the balance between electron donor (HS^−^) and electron acceptor (NO_3_^−^) availability likely plays a key role in determining the end product of NO_3_^−^ reduction, both within and between mat types (Fig. 4). DNRA generates more energy per NO_3_^−^ reduced (26), making it more favorable when plenty of HS^−^ is available. The steeper HS^−^ gradients beneath orange FLSB mats can cause HS^−^ to reach the sediment surface (20), where it may favor the orange filaments that specialize in DNRA over the white filaments that maintain the ability to denitrify as well. On the other hand, denitrification uses electrons more efficiently, requiring only 5 electrons to reduce one NO_3_^−^ compared with 8 electrons for DNRA (27). Therefore, white mats may have an advantage where hydrothermal HS^−^ fluxes are lower and filaments must rely on stored electron donor (S^0^) pools at least some of the time.

This same mechanism may explain the observed spatial separation between denitrification and DNRA within the white FLSB mat (Fig. 4). Denitrification makes efficient use of limited intracellular S^0^ supplies near the surface where NO_3_^−^ is freely available in the seawater medium, while DNRA makes efficient use of limited stored NO_3_^−^ deeper in the mat where abundant HS^−^ is supplied by the sediment. An often-cited alternative explanation is that denitrification is inhibited by HS^−^ (28), making DNRA necessary in its presence. However, Sørensen et al. (28) showed HS^−^ inhibition of only the final nitrous oxide reduction step of denitrification, not the process as a whole. Therefore, denitrification may only be less efficient in the presence of HS^−^, making it more likely to be outcompeted by the more energetically favorable process of DNRA. Indeed, we observed a small amount of denitrification activity (N_2_O accumulation) deep in the acetylene-amended laboratory-incubated white FLSB mat, demonstrating that denitrification can function to a certain extent under sulfidic conditions, though DNRA was clearly the dominant process here (Fig. 2).

Studies that aim to determine the environmental controls on the competition between denitrification and DNRA tend to focus on this competition at the organismic level, where different bacteria carry out each process separately (26, 27). The white FLSB are extremely interesting in this context because the same organism carries out both processes. Therefore, the observed differences in NO_3_^−^ reduction activity could not be caused by between-organism differences in enzymes, metabolic pathways, or other cellular processes. Instead, differences in NO_3_^−^ reduction activity must result from the up- and downregulation of the same enzyme systems within the same organism. Our results suggest that the balance between electron donor and electron acceptor availability controls the switch between the denitrification and DNRA pathways, not just at the level of between-organism competition (26, 27), but also at the even more fundamental level of enzyme system regulation.

## MATERIALS AND METHODS

### Preparation of the filaments.

Samples of white and orange FLSB mats were collected with push cores from hydrothermal sediments of Guaymas Basin in the Gulf of California (Table S1 and Fig. S1) during cruise AT37-06 with R/V Atlantis and deep-sea submersible DSV *Alvin*. Freshly recovered samples were transferred to the ship's cold room (4°C). FLSB mats were gently pulled off the tops of sediment cores using a pipette and placed in 100-ml plastic beakers containing seawater. Some sediment adhered to the filaments that, when left overnight, settled at the bottom of the beaker while the filaments reformed a mat on top of it. The mat was transferred to another beaker containing fresh seawater and allowed to settle again. This process was repeated several times to clean and enrich the filaments as much as possible before they were used for experiments.

### Controlling for FLSB-specific activity.

In spite of this cleaning, a diversity of small microbial cells usually remain adhered to the filaments, as demonstrated previously using a more meticulous cleaning procedure for single filaments (12). To distinguish the activity of the FLSB from the small microorganisms growing on the filaments (epibionts) in this study, we destroyed the large filaments mechanically while leaving the small cells intact. After incubating precleaned intact FLSB mats for ∼20 h with different treatments (see below), the mats were drawn into a 6-ml syringe through a needle (0.41 mm inner diameter, 22 gauge) and reinjected with force into the incubation vial. This step was repeated three times. Microscopic examination of the sample confirmed that the shear forces in the needle destroyed all filaments. Most of the smaller associated cells likely remained intact, as evidenced by the nonzero activities measured in the ∼12 h of incubation following this treatment. In another study (29), scientists also employed a mechanical method (using a Potter-Elvehjem homogenizer) to destroy closely related multicellular “Candidatus Marithioploca” filaments for the same purpose. Using fluorescence microscopy, they observed that the wide filaments were destroyed by this treatment, while the majority of the bacterial epibionts remained intact. By incubating first the intact FLSB mat with its associated bacteria and then the crushed filaments under the same conditions, it was possible to directly compare the NO_3_^−^ reduction activity of the whole mat community with that of the epibionts only, and to determine the contribution of the FLSB filaments by subtraction. This strategy was employed in the two incubation experiments described below.

A separate experiment was performed to determine whether this treatment also kills smaller marine bacteria. Liquid medium (0.2 liters Difco marine broth 2216 culture medium amended with 3.5 g · liter^−1^ NaNO_3_) was inoculated with 0.5 ml of marine sediment and incubated at 25°C for 2 days, from which a fresh bottle of the same medium was reinoculated and incubated for 24 h at 25°C. Six milliliters of this culture was pulled 3 times through a 22-gauge syringe, as described above, and the rest was untreated. From plate counting a dilution series on 1.5% agar plates of the same medium, we found 9.4 × 10^6^ CFU · ml^−1^ in the treated suspension and 6.1 × 10^6^ CFU · ml^−1^ in the untreated suspension. Thus, the shear stress that destroyed the large FLSB filaments was insufficient to kill much smaller (∼1 μm) bacteria similar to the epibionts of the FLSB filaments.

### [^15^N]NO_3_^−^ incubations.

Precleaned white and orange FLSB mats were incubated separately in 50 μM [^15^N]NO_3_^−^ for 3 h, allowing individual filaments to take up the labeled substrate and store it in their vacuoles. The mats were then rinsed twice with ice-cold surface seawater to remove the residual labeled NO_3_^−^. The resulting white and orange mats were each separated into three equal pieces and placed into 60-ml syringes such that each syringe contained 2 to 5 ml of mat material. This was done at two different sampling locations (Table S1 and Fig. S1), so that a total of six orange and six white mat pieces were used in these incubations. Syringes were filled to 60 ml total volume with N_2_-purged surface seawater that was amended with unlabeled NO_3_^−^ to a final concentration of 50 μM. The seawater was aerated during the transfer process such that oxygen concentrations were 100 to 200 μM throughout the incubations. Two mats of each color type were given HS^−^ addition treatments, in which syringes were injected with a pH-neutralized HS^−^ solution to a final concentration of 100 μM. One mat of each color type was given an acetate addition treatment, in which a pH-neutralized acetic acid solution was added to each syringe to a final concentration of 375 μM. Syringes were sealed with a three-way valve and incubated at 4°C in the dark. The small number of treatment replicates was necessitated by a shortage of scientific supplies available on the ship caused by customs problems in the host country. These treatments were intended to illustrate whether both NO_3_^−^ reduction pathways were active across a range of seawater chemistries, not to determine whether altering electron donor availability resulted in statistically significant differences in process rates.

Subsamples from each 60-ml syringe were collected after 0, 2.5 to 3.5, 7.5 to 8.5, and 18 to 20 h of incubation. Prior to subsampling, the incubation syringes were mixed by inverting several times and then left to settle upright with the three-way valve on top. Once the filaments had settled on the piston end, a long needle was attached to the three-way valve perpendicular to the syringe, and 10.5 ml of the overlying seawater was injected through this needle into the bottom of a 12-ml Exetainer (Labco, UK) containing 1.5 ml of a saturated zinc acetate solution as fixative. During the first phase of the incubations with the intact FLSB mats, all filaments stayed in the 60-ml syringe during subsampling. The Exetainers were filled completely with no headspace, sealed, and inverted several times to stop all activity. After the final subsampling, the remaining seawater medium was removed until the FLSB filaments remained in a minimal volume of 5 ml, before crushing the large FLSB filaments as described above. Of the resulting suspension, 500 μl was pipetted into a microcentrifuge tube containing 150 μl of 6 N HCl and stored at 4°C for protein analysis. The 60-ml syringes were refilled with seawater medium, and the chemistry was adjusted as described above to match the initial incubation conditions in each syringe. Four additional subsamples were collected at 20 to 22.5, 22 to 24, 27.5 to 29.5, and 31 to 33 h of total incubation time.

The concentrations of argon, O_2_, ^28^N_2_, ^29^N_2_, and ^30^N_2_ in the Exetainers were measured using a membrane inlet mass spectrometer (MIMS; GAM200, IPI). The MIMS signals were drift-corrected and calibrated using instrument blank and aerated seawater signals. Excess ^29^N_2_ and ^30^N_2_ concentrations were calculated relative to air as previously described (30). The total excess ^15^N concentration was calculated according to the equation

[N15]ex=[N229]ex+2[N230]ex

Argon concentrations tended to increase steadily throughout the incubations, indicating gas diffusion across the polyethylene syringe wall. This leak also resulted in a net loss of [^15^N]N_2_ when denitrification activity stopped. To account for this loss, the total excess ^15^N concentrations were corrected for changes in argon concentration relative to the apparent argon saturation concentration ([Ar]_app_) according to the equation

[N15]Ar=[N15]ex([Ar]app−[Ar]min)/([Ar]app−[Ar]i)

where [^15^N]_Ar_ is the argon-corrected total excess ^15^N concentration, [^15^N]_ex_ is the total excess ^15^N concentration calculated according to equation 8, [Ar]_min_ is the minimum argon concentration measured during a set of samples, and [Ar]_i_ is the argon concentration measured at the time point for which the correction is being calculated. [Ar]_app_ was estimated to be 9.4 μM by finding the highest value that compensated for the maximum observed ^15^N loss. In general, this correction had a minor effect on the higher denitrification rates with the intact FLSB filaments but accounted for the net loss of [^15^N]N_2_ after destruction.

Following MIMS analysis, subsamples were treated with hypobromide to convert [^15^N]NH_4_^+^ into ^29^N_2_ and measured on a gas chromatogram-isotope ratio mass spectrometer (GC-IRMS) (30). Conversion was consistently greater than 95%. Excess ^29^N_2_ and ^30^N_2_ concentrations were calculated relative to air, and the total excess ^15^N concentration was calculated according to equation 8, as described above.

Protein content was measured using a Pierce BCA protein assay kit-reducing agent compatible (catalog no. 23250; Thermo Fisher). The change in [^15^N]N_2_ and [^15^N]NH_4_^+^ concentrations between each pair of time points ([^15^N]_ti_ − [^15^N]_ti__−__1_) was calculated and corrected for the changing incubation volume (*V*, in liters) and protein content (*P*, in micrograms of protein), according to the equation

ΔN15=([N15]ti−[N15]ti − 1)×1,000×V/P

to produce Δ^15^N (nanomoles ^15^N per microgram of protein), the volume- and protein-normalized change in ^15^N content. Total protein content was used as a proxy for mat biomass in these incubations. Following filament destruction, the mat was homogenized throughout the seawater medium, resulting in biomass loss due to subsampling. Therefore, rates were normalized to protein content to account for this loss of mat biomass. Rates of [^15^N]N_2_ and [^15^N]NH_4_^+^ production were calculated as cumulative Δ^15^N versus time. The time point immediately following filament destruction was not used in rate calculations because concentrations were often disproportionately high relative to the following samples, possibly due to incomplete mixing with the residual ^15^N from the first part of the incubation or due to carryover in the sample needle. Due to the uncertainties related to the calculation of overall denitrification and DNRA rates following the random isotope pairing principle (31) in the presence of considerable intracellular storage of nitrate (32), we only report the ^15^N products in this study. Hence, the overall denitrification and DNRA rates per protein could be several times higher than the ∼10 to 1,000 pmol N · (μg protein)^−1^ · h^−1^ reported here.

### Microsensor measurements.

A batch of white FLSB filaments survived the journey to the laboratory in Bremen in a 100-ml plastic screw-cap cup. The filaments had formed a 2-cm-thick mat, which was used for microsensor measurements. The temperature was maintained at 2°C, and the seawater overlying the mat was very gently aerated and mixed via airflow through a Pasteur pipet (one bubble every 2 to 3 s). Every other day, the water column was refreshed with artificial seawater (salinity, 3.5%) containing 50 μM NO_3_^−^. Microsensors for O_2_, N_2_O, and pH were constructed and calibrated as described previously (33–35). The tip diameters were 10 μm for the O_2_ and pH sensors and 20 μm for the N_2_O sensor. Measurements with the three sensors were done simultaneously. The depth at which each sensor touched the mat surface was observed using a dissection microscope and carefully noted for later alignment of the profiles. Experiments with acetylene, which inhibits N_2_O reductase (36) and allows N_2_O to accumulate during denitrification, were performed by replacing 20% of the seawater medium with the same medium that had been saturated with acetylene (washed to remove acetone). The overlying seawater was not aerated or mixed during the acetylene experiment.

### Phylogeny.

The phylogenetic tree of Guaymas Beggiatoaceae was calculated in Arb by neighbor joining with the Felsenstein distance correction (37, 38) and 1,000 bootstrap replicates. Only complete or near-complete sequences were used and then filtered to include only positions with information for all species and remove introns; the final alignment included 1,401 positions. The 16S rRNA genes for the orange Beggiatoaceae group include IMG locus tags BOGUAY_3612 and Ga0193933_102039 from a metagenome-reconstructed genome (16), PCR amplicons with GenBank accession numbers JN793553 and JN793556.1 from individual cleaned filaments (20), and an amplicon with GenBank accession number KP091103 from mat-covered sediment (39). The accession numbers for the large white Beggiatoaceae include IMG locus tag Ga0193912_101714 from a metagenome-reconstructed genome (16), PCR amplicons with GenBank accession numbers JN793554.1 and JN793557 from individual cleaned filaments (20), and an amplicon with GenBank accession number KJ569660 from mat-covered sediment (40). Four Desulfovibrio sequences were used to root the tree. The incomplete 16S rRNA sequence of Beggiatoaceae bin 4572_84 (Ga0193912_101714) (16) is 100% identical to three of the four sequences in the group of other wide white FLSB over its 384-nucleotide length and affiliates with them in trees calculated from truncated 16S rRNA gene sequences (not shown).

### Genomic analysis.

To evaluate whether denitrification and DNRA can cooccur in the same FLSB, the following publicly available genomes from the family Beggiatoaceae were gathered: orange-pigmented and unpigmented (“wide white”) Guaymas Beggiatoaceae, “Candidatus Thiomargarita nelsonii,” “Candidatus Marithrix,” Thioploca ingrica, Beggiatoa alba, and Beggiatoa leptomitiformis. These were considered complete enough (>85% genome completeness) to assess their NO_3_^−^ reduction pathway genes. Gene candidates were identified by keyword and BLASTP searches of IMG/ER (41).

The two Guaymas Basin data sets (orange and white filaments) were produced from microbial mats overlying hydrothermally influenced sediments in Guaymas Basin (Mexico). The bin ex4572_84_Beggiatoa genome (wide white filaments) was assembled from a sediment metagenome from combined 0- to 3-cm and 12- to 15-cm-depth intervals and is estimated to be 86% complete (16) (Fig. 3). The “Orange Guaymas” sequence was obtained from a single cleaned filament (13, 23) and is estimated to be 98% complete (18). Beggiatoa alba B18LD^T^ is a nonvacuolated strain isolated from freshwater rice paddy sediment (42, 43). Its genome is estimated to be 100% complete by single-copy gene complement (44) but is not closed, consisting of one long and two short linear contigs. Thioploca ingrica grows as sheathed trichomes (filaments) lacking large vacuoles; the genome sequence was obtained from the metagenome of multiple trichomes collected from Lake Okotanpe (Japan) sediment (14) and is closed. The “*Ca*. Thiomargarita nelsonii” bud S10 genome was obtained from a budding vacuolated cell attached to a gastropod shell at the Hydrate Ridge methane seep (OR, USA) (44) and is estimated to be 87% complete. The “*Ca*. Marithrix” Green Canyon 246 genome was sequenced from a single filament (cut into segments) collected from sediments near deep-sea brines in the Gulf of Mexico and is estimated to be 94% complete (15).

## Supplementary Material

Supplemental material
