# Supplementary material for: Filamentous Giant Beggiatoaceae from the Guaymas Basin Are Capable of both Denitrification and Dissimilatory Nitrate Reduction to Ammonium
Source: Appl Environ Microbiol. 2018 Jul 17;84(15):e02860-17. doi: 10.1128/AEM.02860-17 (PMC6052272; doi:10.1128/AEM.02860-17)
Supplement: Supplemental material [file supp_84_15_e02860-17__index.html]

Filamentous Giant Beggiatoaceae from the Guaymas Basin Are Capable of both Denitrification and Dissimilatory Nitrate Reduction to Ammonium — Supplemental material 

# Filamentous Giant Beggiatoaceae from the Guaymas Basin Are Capable of both Denitrification and Dissimilatory Nitrate Reduction to Ammonium

## Supplemental material

- Supplemental file 1 -

  Supplemental methods, results, and discussion; sampling dates and locations of the cores from which FLSB mats were collected (Table S1); sampling sites for orange and white FLSB mats (Fig. S1); ORF designations (Table S2); NarH candidates (Fig. S2); inferred phylogeny, gene neighborhoods, and domain structure for the NarH-like predicted protein in *Thioploca ingrica* (Fig. S3); N2O production (Fig. S4).

  PDF, 1.4M
