# Supplementary material for: Filamentous Giant Beggiatoaceae from the Guaymas Basin Are Capable of both Denitrification and Dissimilatory Nitrate Reduction to Ammonium
Source: Appl Environ Microbiol. 2018 Jul 17;84(15):e02860-17. doi: 10.1128/AEM.02860-17 (PMC6052272; doi:10.1128/AEM.02860-17)
Supplement: Supplemental material [file AEM.02860-17_zam015188652s1.pdf]

Supplementary Methods

Porewater analysis – Porewaters from the Guaymas Basin were analyzed for nitrate+nitrite (NO_x) and NH_4^+ concentrations colorimetrically using a Flow Solutions IV segmented flow Auto Analyzer from O.I Analytical, College Station, TX. The HCl-acidified samples were neutralized with NaOH before analysis. NO_x was determined using the cadmium reduction method and NH_4^+ was determined using the phenate method. Both nutrients were diluted to get their concentrations within the linear range of the auto analyzer. Quality control standards from certified stock standard purchased from Environmental Research Associates, were analyzed every 15-20 samples.

Fluorescent staining – Filaments from a white mat were removed with a plastic pasteur pipette and transferred into a petri dish with cold artificial seawater, moved around to remove most sediment debris attached to the sheath, and transferred to a glass petri dish containing 2% formaldehyde in artificial seawater. They were fixed for 30 min at room temperature, and washed 3x with artificial seawater. After removing most of the liquid, 1 ml of staining mix was added (0.1 mg/ml fluorescein isothiocyanate (FITC, Thermo Fisher Scientific) and 8 μM Nile Red (Sigma Aldrich); final concentrations in PBS) and incubated for 1 hour at room temperature. Filaments were carefully transferred onto a microscope slide containing a frame made of electrical tape as a space holder to not crush the filaments when the cover slip is applied. Images were taken with the confocal laser scanning microscope LSM 780 (Zeiss, Jena, Germany) using the 488 nm and 561 nm laser.

Scanning electron microscopy – White, wide filaments were sampled, washed, and fixed as described for fluorescent staining. Post-fixing, they were washed 3x in

MilliQ water. Then, filaments were placed onto poly-lysine-covered silica wafers and allowed to settle. Gradually, the water was removed by adding and carefully removing ethanol of the concentrations 30%, 50%, 70%, 80%, and 96%, without letting the sample run dry. In a critical point dryer (EM CPD300, Leica Microsystems, Wetzlar, Germany) the ethanol was replaced by liquid CO_2, which was then let evaporate. The dried wafers were placed onto a carbon tape on a specimen stub. To break the filaments a second tape-covered specimen stub was carefully placed on top of the filament-containing one and removed again. Samples were sputtered with carbon (EM ACE600, Leica Microsystems, Wetzlar, Germany). Images were taken with a Quanta250 scanning electron microscope (FEI, Oregon, USA) using 2 kV.

N_2O production rates – White FLSB filaments were collected from cores 4862-02 and 4862-04. Filaments were placed in a cup of seawater overnight and allowed to form a mat. 40 mL of N_2-purged surface seawater was added to six 50-mL centrifuge tubes. The white mat was divided evenly between two centrifuge tubes, representing 5-10% (3-5 mL) of the total incubation volume. Tubes were filled to 50 mL total volume with 10 mL of acetylene-purged seawater. All tubes were amended with 50 µM NO_3^- and sealed with plastic wrap to limit gas exchange. Mats were incubated at 4°C with occasional mixing by inversion. Each tube was sampled at 0, 0.5, 1.5, 3.5, 6, 7, 9, and 11 hours following the start of incubation. After the 6-hour time point, the FLSB filaments were destroyed as described in the main text. Subsamples were collected by gently mixing and transferring 2 mL liquid, into a 1.5 mL Eppendorf tube containing 25 microliters of 6N HCl. The Eppendorf tubes were sealed tightly with no headspace and stored under oil to prevent gas exchange. Upon return to the laboratory, 1 mL of each

sample was injected into a He-purged 3 mL exetainer and shaken overnight. The concentration of N_2O in the headspace was measured on a gas chromatograph with electron capture detector.

Supplementary Results and Discussion

***Beggiatoaceae* nitrate reductases** – The candidate NarH gene found in *Ca*. Marithrix (Fig. S2A) was phylogenetically distinct from the tight cluster of candidate NarH genes found in the other *Beggiatoaceae* (except *Thioploca ingrica*; Fig. S2B); the different phylogenetically mixed closest neighbors of each group suggest these nitrate reductases may have been acquired in two separate horizontal transfer events. In all cases, these predicted NarH genes were located within putative Nar operons and annotated as nitrate reductase subunits in CDD. No canonical NarH gene was found in the *Thioploca ingrica* genome; the ORF described as NarH by Kojima et al. (1) is closely related to ORFs in other *Beggiatoaceae* (Fig. S3) that are classified as DMSO reductases in IMG, and selenate reductases in CDD. Experimental evidence will be needed to clarify the physiological substrate(s) of these predicted proteins.

Supplementary Figures and Tables

Table S1. Sampling dates and locations of the cores from which FLSB mats were collected. Asterisks indicate cores used for porewater NO_x and NH_4^+ analysis.

**Location**	**Latitude/ Longitude**	**Sampling Date**	**Dive #**	**Mat**	**Core #**
Ultra Mound	27°00.440 N 111°24.528 W	Dec. 19 2016	4868	White	7, 8
Ultra Mound	27°00.445 N 111°24.535 W	Dec. 21 2016	4869	White	1
				Orange	3, 16*, 17, 19, 21*, 26, 29
Cathedral Hill	27°00.680 N 111°24.270 W	Dec. 24 2016	4872	White	3, 6*, 12, 22, 23, 24
				Orange	14*, 15, 17

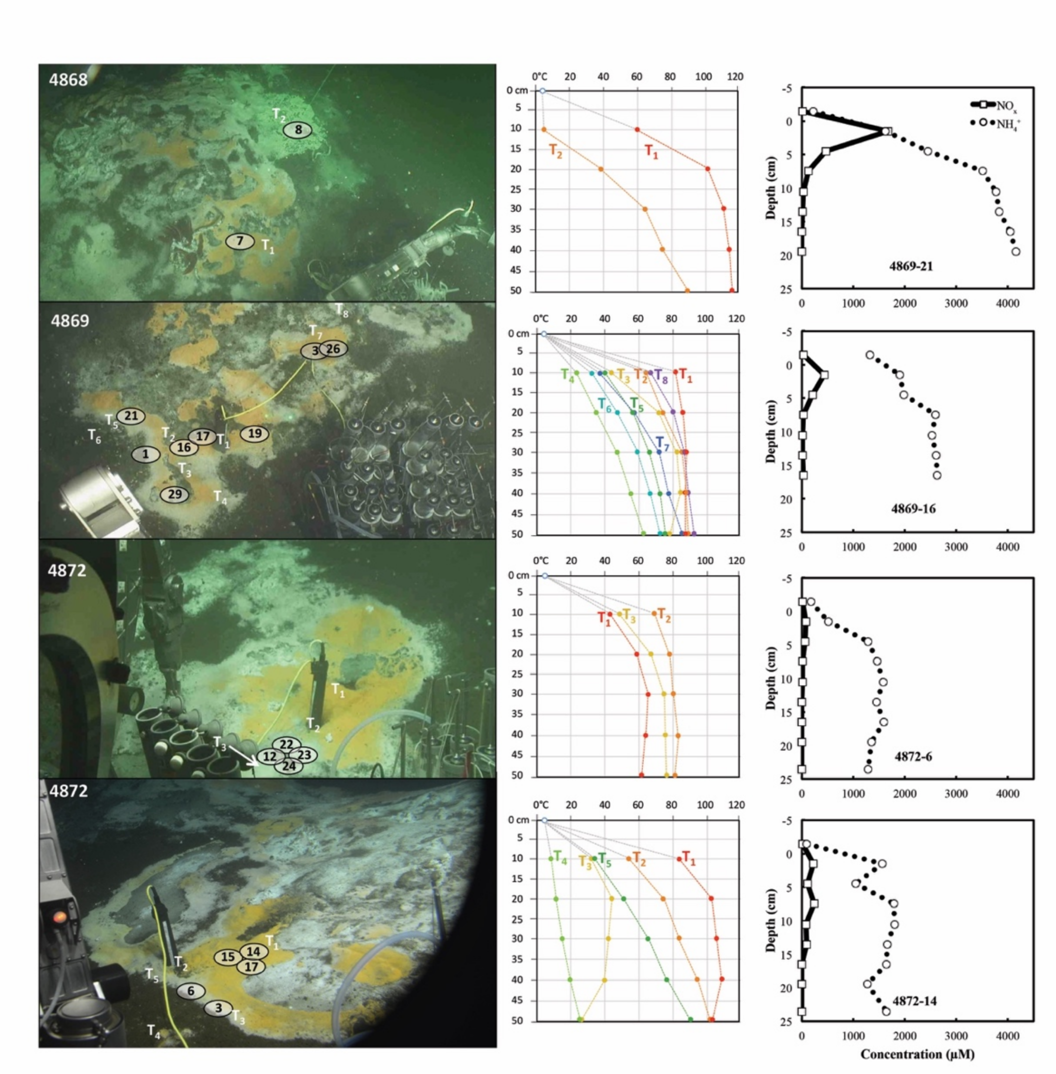

Figure S1. Sampling sites for orange and white FLSB mats as listed in Table S1, showing locations of sediment cores for mat collection, porewater NO_x and NH_4^+ analysis, and temperature profiles determined with the *Alvin* heatflow probe (2). Temperature loggers were spaced every 10 cm along the heatflow probe; measurement points started at 10 cm depth when the probe is fully inserted into the sediment. Thermal readings were taken after several minutes to allow the profiles to stabilize. The surface temperature of 3°C is the temperature of Guaymas Basin bottom water.

Table S2. ORF designations for the *Beggiatoaceae* NO_3^- reduction pathway genes displayed in Table 1. Gene candidates were identified by key word and BLASTP searches of IMG/ER (3). TAT signal peptides were predicted using the TatP 1.0 Server (4). CDD, Conserved Domain Database (5). Diversity in predicted NO_3^- respiration pathways is widespread among members of the *Beggiatoaceae*. As previously described (6), the large vacuolated chain-forming *Ca.* Thiomargarita nelsonii has complete sets of predicted genes for both DNRA and denitrification, with the caveat that NirM was not positively identified. Narrow unvacuolated freshwater *Thioploca ingrica* (1) likewise may have the potential for both pathways, although some subunits of its candidate NarG are affiliated with possible DMSO rather than NO_3^- reductases (Fig. S2B) Large vacuolated filamentous *Ca.* Marithrix sessilis (7) appears capable of DNRA only, lacking genes for all but a few accessory denitrification pathway proteins (NirE, NorD, NorQ). The freshwater *B. alba* is distinct from all of these, as expected from experimental observations (8): putative genes were found for the non-respiratory periplasmic nitrate reductase NapA, and for nitrite reduction to ammonium, but not for denitrification.

**Activity**	**Proteins**	**Description**	**"Beggiatoa" sp. Orange Guaymas**	**bin ex4572_84_"Beggiatoa" (wide white Guaymas)**	***Ca.* Thiomargarita nelsonii bud S10**	***Thioploca ingrica***	***Ca.* Marithrix sp.Green Canyon 246**	***Beggiatoa alba* B18LD**
**Nxr/Nar respiratory nitrate reductase**	**NarG/NxrA[1]**	**Alpha subunit**	BOGUAY_0489	Ga0123547_109421	Ga0063879_01731, Ga0063879_03576	not found	Ga0199145_101135	not found
	**NarH**	**Beta subunit**	BOGUAY_0490	Ga0123547_109420	Ga0063879_01732, Ga0063879_03575	not found	Ga0199145_101136	not found
	**NarJ**	**Molybdenum**	BOGUAY_0491	Ga0123547_109418	Ga0063879_01733	not found	Ga0199145_101137	not found

		**cofactor chaperone**						
	**NarI**	**Gamma subunit**	BOGUAY_0492	Ga0123547_109417	Ga0063879_01734, Ga0063879_03573	not found	Ga0199145_101138	not found
**Nar-like reductase**	**NarG (TAT signal peptide)[1]**	**Alpha subunit**	BOGUAY_0051/ BOGUAY_0050	Ga0123547_10332	not found	Ga0060138_113819	not found	not found
	**NarH**	**Beta subunit**	BOGUAY_0049	Ga0123547_10336	not found	Ga0060138_113818	not found	not found
	**NarJ?**	**DMSO reductase family type II enzyme, heme *b* subunit**	BOGUAY_0048	Ga0123547_10337	not found	Ga0060138_113817	Ga0199145_106186?	not found
	**NarI**	**Chaperone TorD involved in molybdoenzyme TorA maturation**	BOGUAY_0046	Ga0123547_103311	not found	Ga0060138_113816	not found	not found
**Periplasmic nitrate reductase**	**NapF**	**Ferredoxin-type protein**	BOGUAY_5179	Ga0123547_100142	Ga0063879_03478	not found	not found	BegalDRAFT_2363
	**NapD**	**Chaperone**	not found	Ga0123547_100141	Ga0063879_03475	not found	not found	BegalDRAFT_2364
	**NapA**	**Large subunit**	BOGUAY_0671	Ga0123547_100140, Ga0123547_13223? (has TAT signal)	Ga0063879_03474	Ga0060138_113718	not found	BegalDRAFT_2365
	**NapG**	**Ferredoxin-type protein**	not found	Ga0123547_12978, Ga0123547_13224	Ga0063879_03473	Ga0060138_112596 (with nitrous oxide	not found	BegalDRAFT_2366

						reductase proteins)		
	**NapH**	**Ferredoxin-type protein**	not found	Ga0123547_12976, Ga0123547_12977 (partial), Ga0123547_13225	Ga0063879_03282, Ga0063879_03468	Ga0060138_112595 (with nitrous oxide reductase proteins)	not found	BegalDRAFT_2367
	**NapB**	**Cytochrome *c*-type protein**	BOGUAY_0672	Ga0123547_100139	Ga0063879_03467	not found	not found	BegalDRAFT_2368
	**NapC**	**Periplasmic subunit**	BOGUAY_3223	Ga0123547_122612	Ga0063879_00366, Ga0063879_03466	Ga0060138_113800 (upstream of two annotated sulfide dehydrogenase proteins)	not found	BegalDRAFT_2369
**Nitrite reductase (NADH)**	**NirB**	**Large subunit**	not found	Ga0123547_12541, Ga0123547_13111 (probably two parts of same gene, 13111 is downstream; may be transposon in between)	Ga0063879_01287	Ga0060138_113714	Ga0199145_102221	BegalDRAFT_1383, BegalDRAFT_3410 (by Conserved Domain Database)
	**NirD[2]**	**Small subunit**	not found	Ga0123547_13112	Ga0063879_01288	Ga0060138_113715	Ga0199145_102220	BegalDRAFT_1382?
**Nitrite reductase (NO-forming)**	**NirS**	**Nitrite reductase (NO-forming) / Hydroxylamine reductase [EC:1.7.2.1 1.7.99.1] (nirS), cyt cd_1 type**	BOGUAY_2967	Ga0123547_100144, Ga0123547_13265, Ga0123547_13266	Ga0063879_04847	Ga0060138_112610	not found	not found

	**NirM**		**Omitted here because various cytochrome types are found in this position in the putative operons, not sure which one(s) function in nitrite reduction**					
	**NirC**	***c*-type cytochrome *c_{55x}***	not found	Ga0123547_13264 ? (right position but different cytochrome type)	Ga0063879_ 04851	Ga0060138_ 112609	not found	not found
	**NirF**	**Periplasmic; NirS maturation**	not found	Ga0123547_13263	Ga0063879_ 04852	Ga0060138_ 112608	not found	not found
	**NirD[2]**	**Siroheme decarboxylase**	not found	Ga0123547_13262	Ga0063879_ 04853	Ga0060138_ 112607	not found	not found
	**NirL**	**Siroheme decarboxylase**	not found	Ga0123547_13986	Ga0063879_ 04854	Ga0060138_ 112606	not found	not found
	**NirG**	**Siroheme decarboxylase**	not found	Ga0123547_13985	Ga0063879_ 04855	Ga0060138_ 112605	not found	not found
	**NirH**	**Siroheme decarboxylase**	not found	Ga0123547_13983	Ga0063879_ 04856	Ga0060138_ 112604	not found	not found
	**NirJ**	**Heme *d_1* biosynthesis radical SAM protein**	not found	Ga0123547_13981	Ga0063879_ 04857	Ga0060138_ 112603	not found	not found
	**NirE**	**Uroporphyrinogen III methyltransferase**	BOGUAY_ 0877	Ga0123547_10425	Ga0063879_ 05504	Ga0060138_ 11548	not found	not found
**Nitric oxide reductase**	**NorB**	**Large subunit**	BOGUAY_ 0863	Ga0123547_112034, Ga0123547_119226	Ga0063879_ 02916	Ga0060138_ 111184	not found	not found
	**NorC**	**Small subunit**	BOGUAY_ 4015	Ga0123547_112035, Ga0123547_119225	Ga0063879_ 02915	Ga0060138_ 111185	not found	not found

	**NorD**	**Activation protein**	not found	Ga0123547_10746, Ga0123547_112036	Ga0063879_04846	Ga0060138_111180	Ga0199145_10739	BegalDRAFT_2688
	**NorE**		not found	Ga0123547_112033	Ga0063879_02934	not found	not found	not found
	**NorQ**		not found	Ga0123547_112031, Ga0123547_12333, Ga0123547_13202	Ga0063879_02936, Ga0063879_02939, Ga0063879_04308	Ga0060138_111183	Ga0199145_10740	BegalDRAFT_1532
**Nitrous oxide reductase**	**NosD**	**Copper insertion**	not found	Ga0123547_12979	Ga0063879_03285	Ga0060138_112597	not found	not found
	**NosZ**	**Catalytic subunit**	not found	Ga0123547_11451	Ga0063879_04858	Ga0060138_112601	not found	not found
	**NosL**	**Copper chaperone**	not found	Ga0123547_12973	Ga0063879_03264, Ga0063879_02838	Ga0060138_112593	not found	not found

[1] Predicted gene sequences suggest two forms of NarG in some species. The second one has predicted (4) signal sequences for the twin arginine translocation (TAT) pathway. If these are functional in export, this is unexpected in a cytoplasmic nitrate reductase, and suggests some alternate role for these proteins.

[2] Genes in two different pathways have been designated "*nirD*".

A) NarH candidate, *Ca.* Marithrix

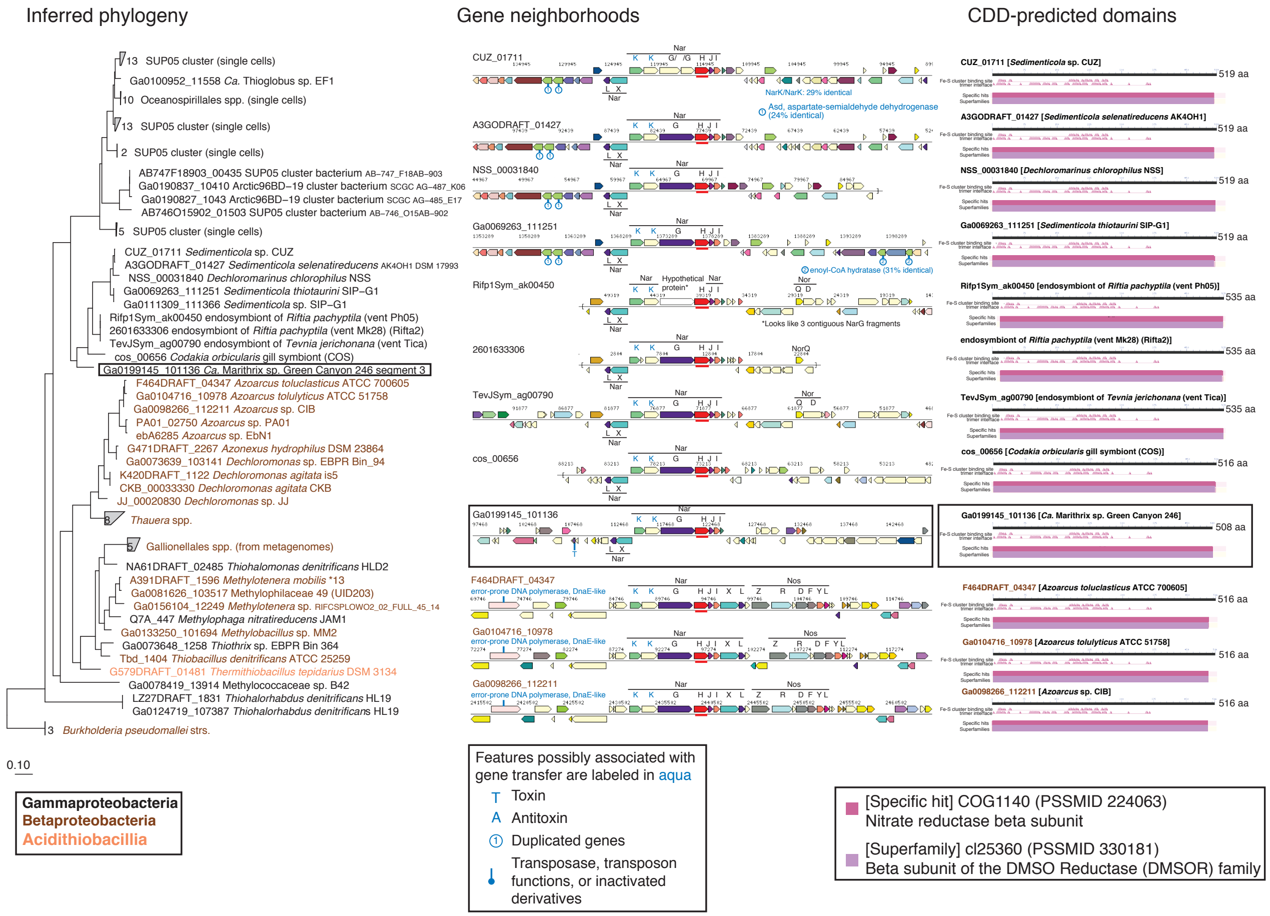

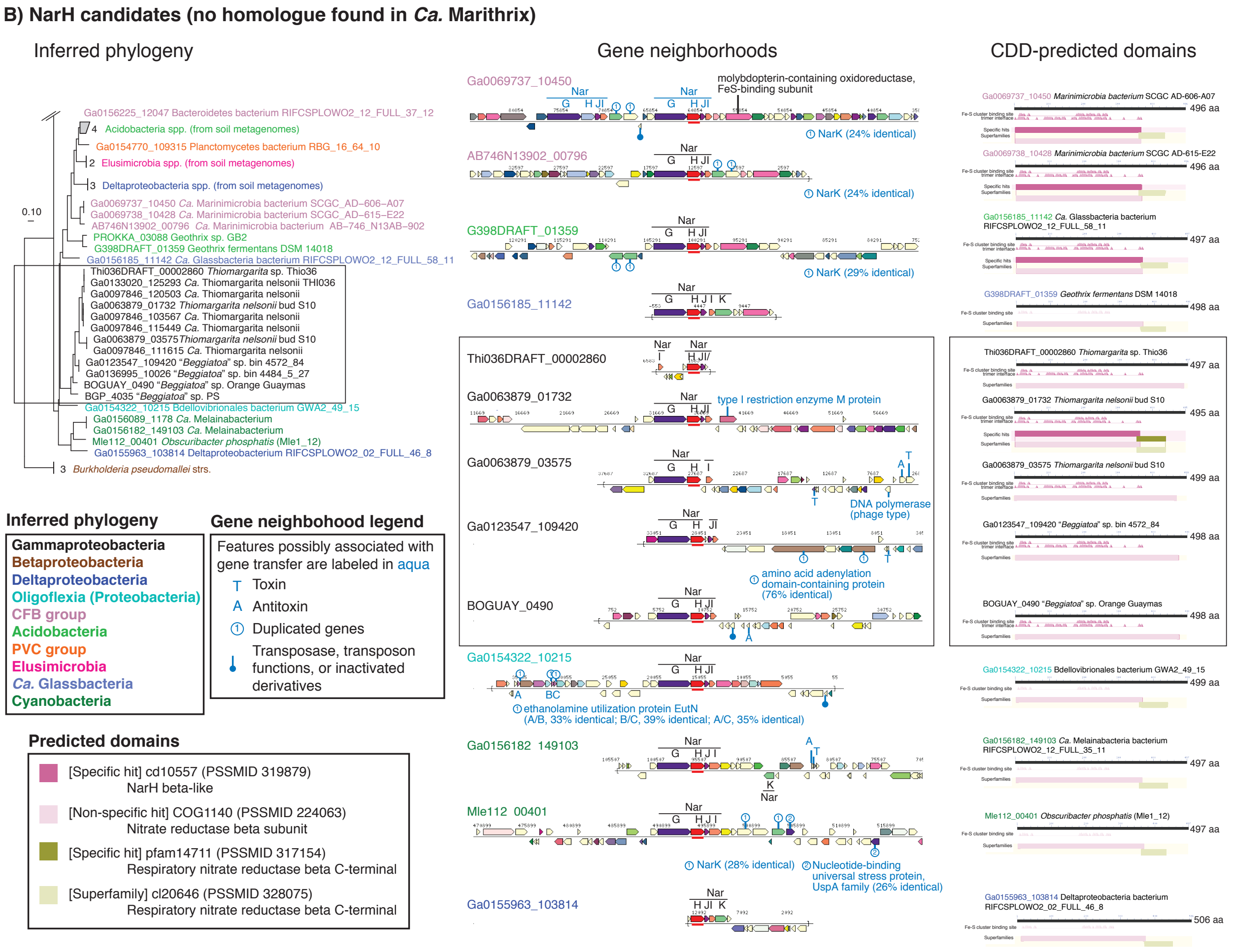

Figure S2. A) NarH candidate from Ca. Marithrix Green Canyon 246 (segment 3). B) NarH candidates from other *Beggiatoaceae*. Protein sequences were selected by BLASTP searches of the IMG/ER database (3) with all annotated Beggiatoaceae NarH candidates; the collected

sequences aligned using MUSCLE (9) in MEGA7 (10), with minor adjustments to the alignments made manually; and neighbor joining used to select closest relatives for subtrees. The final trees were produced using RAxML rapid bootstrapping (11) as implemented in ARB (12), with a random initial tree, the PROTGAMMA rate distribution and WAG amino acid substitution models, empirical amino acid frequencies, and branch optimization. The tree shown was the best of 25 runs. Gene neighborhoods are from IMG/ER (3). Full-length segments are 50 kb long (maximum), centered on the putative NarH genes. Predicted domain structures are from the Conserved Domain Database (5) (the concise view is shown). Only full-length or near full-length sequences were included in the tree, and in most cases gene neighborhoods are shown only for relatively long contigs; an exception was made to show the *Ca.* Thiomargarita neighborhoods in Fig. S3.

NarH-like candidates: Respiratory nitrate reductase beta subunit (IMG annotation)/ Selenate reductase beta subunit (CDD)

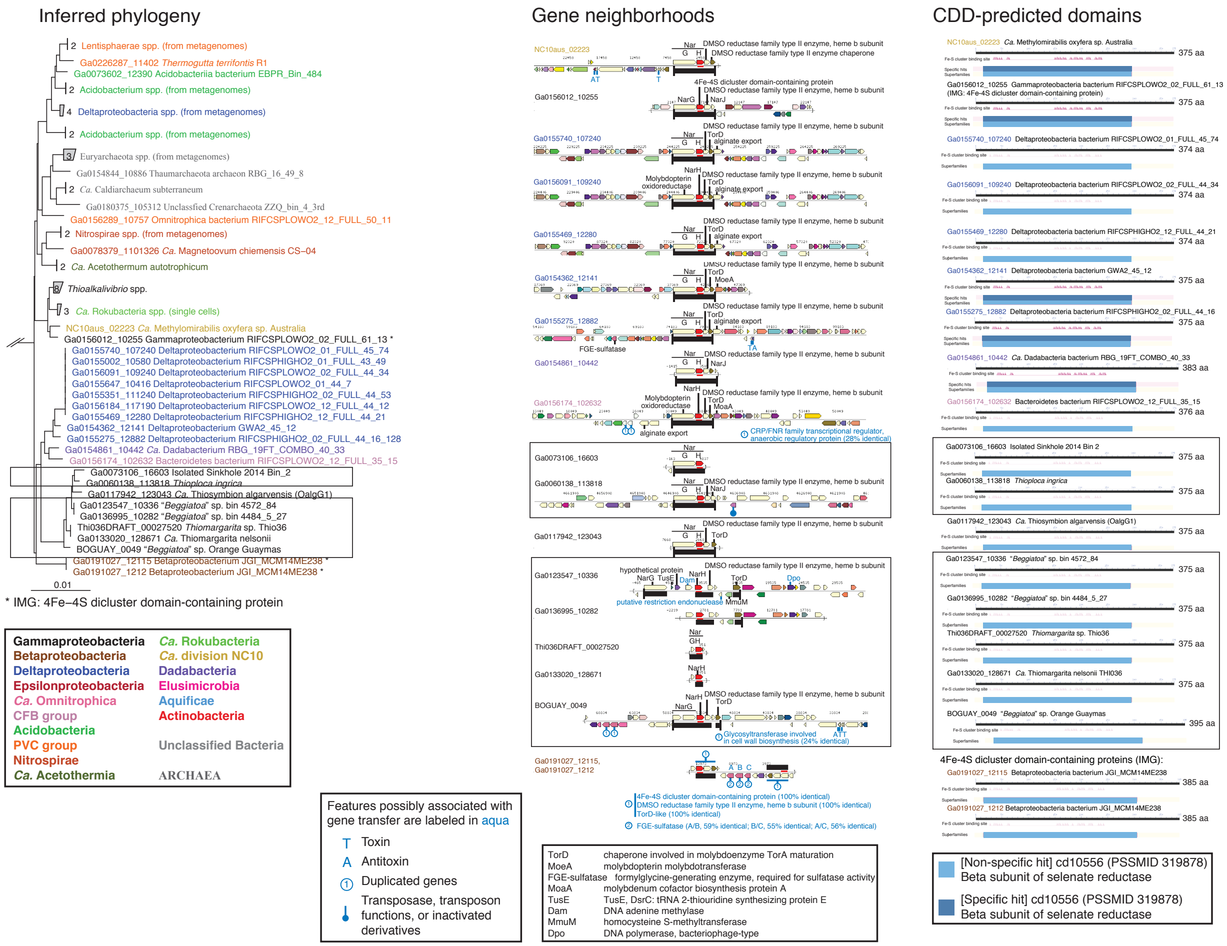

Figure S3. Inferred phylogeny, gene neighborhoods, and domain structure for the NarH-like predicted protein in *Thioploca ingrica*. See the Fig. S2 caption for methods.

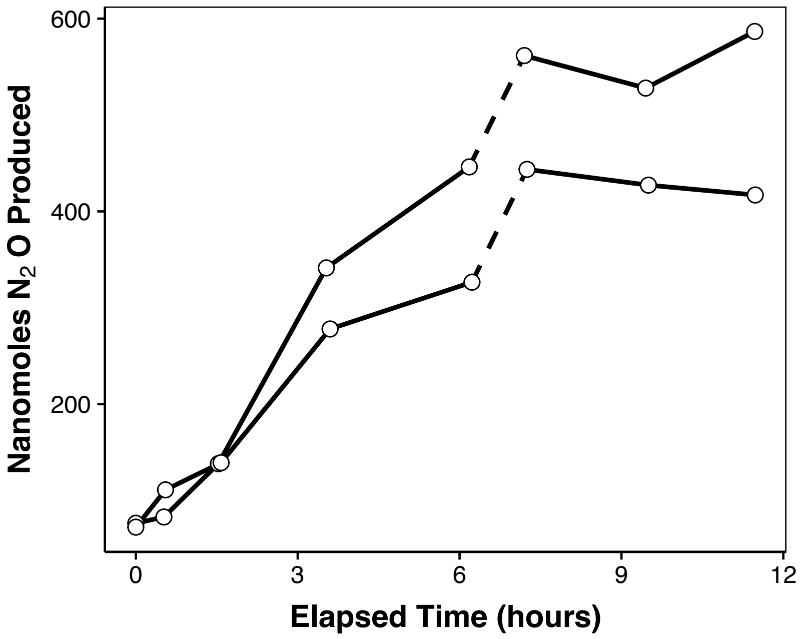

Figure S4. N_2O production in two different white FLSB mats following the addition of 20% acetylene to the seawater medium. The dashed line after ca. 6 hours represents the time point when the FLSB were mechanically destroyed before the incubation was continued.

Supplementary References

1. Kojima H, Ogura Y, Yamamoto N, Togashi T, Mori H, Watanabe T, Nemoto F, Kurokawa K, Hayashi T, Fukui M. 2014. Ecophysiology of *Thioploca ingrica* as revealed by the complete genome sequence supplemented with proteomic evidence. ISME J 9:1166-1176.
2. McKay LJ, MacGregor BJ, Biddle JF, Albert DB, Mendlovitz HP, Hoer DR, Lipp JS, Lloyd KG, Teske AP. 2012. Spatial heterogeneity and underlying geochemistry of phylogenetically diverse orange and white *Beggiatoa* mats in Guaymas Basin hydrothermal sediments. Deep Sea Res Part 1 Oceanogr Res Pap 67:21-31.
3. Markowitz VM, Mavromatis K, Ivanova NN, Chen I-MA, Chu K, Kyrpides NC. 2009. IMG ER: a system for microbial genome annotation expert review and curation. Bioinformatics 25:2271-2278.
4. Bendtsen JD, Nielsen H, Widdick D, Palmer T, Brunak S. 2005. Prediction of twin-arginine signal peptides. BMC Bioinformatics 6:167.
5. Marchler-Bauer A, Lu SN, Anderson JB, Chitsaz F, Derbyshire MK, DeWeese-Scott C, Fong JH, Geer LY, Geer RC, Gonzales NR, Gwadz M, Hurwitz DI, Jackson JD, Ke ZX, Lanczycki CJ, Lu F, Marchler GH, Mullokandov M, Omelchenko MV, Robertson CL, Song JS, Thanki N, Yamashita RA, Zhang DC, Zhang NG, Zheng CJ, Bryant SH. 2011. CDD: a Conserved Domain Database for the functional annotation of proteins. Nucleic Acids Res 39:D225-D229.
6. Flood BE, Fliss PS, Jones DS, Dick GJ, Jain S, Kaster A-K, Winkel M, Mußmann M, Bailey J. 2016. Single-cell (meta-)genomics of a dimorphic *Candidatus* Thiomargarita nelsonii reveals genomic plasticity. Front Microbiol 7:603.
7. Salman-Carvalho V, Fadeev E, Joye SB, Teske A. 2016. How clonal is clonal? Genome plasticity across multicellular segments of a “*Candidatus* Marithrix sp.” filament from sulfidic, briny seafloor sediments in the Gulf of Mexico. Front Microbiol 7:1173.
8. Vargas A, Strohl WR. 1985. Utilization of nitrate by *Beggiatoa alba*. Arch Microbiol 142:279-284.
9. Edgar RC. 2004. MUSCLE: multiple sequence alignment with high accuracy and high throughput. Nucleic Acids Res 32:1792-1797.
10. Kumar S, Stecher G, Tamura K. 2016. MEGA7: Molecular Evolutionary Genetics Analysis Version 7.0 for bigger datasets. Mol Biol Evol 33:1870-1874.
11. Stamatakis A. 2006. RAxML-VI-HPC: Maximum likelihood-based phylogenetic analyses with thousands of taxa and mixed models. Bioinformatics 22:2688-2690.
12. Ludwig W, Strunk O, Westram R, Richter L, Meier H, Yadhukumar, Buchner A, Lai T, Steppi S, Jobb G, Förster W, Brettske I, Gerber S, Ginhart AW, Gross O, Grumann S, Hermann S, Jost R, König A, Liss T, Lüßmann R, May M, Nonhoff B, Reichel B, Strehlow R, Stamatakis A, Stuckmann N, Vilbig A, Lenke M, Ludwig T, Bode A, Schleifer K-H. 2004. ARB: a software environment for sequence data. Nucleic Acids Res 32:1363-1371.
